# Society Proceedings

**Published:** 1899-02

**Authors:** 


### SOCIETY PROCEEDINGS.

### SCHUYLKILL VALLEY VETERINARY MEDICAL ASSOCIATION.

The annual meeting was held at Reading, Pa., December 21, 1898. President Noack called the meeting to order at 11.30 A.M. A large number of members were in attendance.

The President addressed the meeting on "How Necessary it is for All of the Members to be Present." Dr. Daniel Kohler, of Kutztown, Pa., was elected to membership.

Papers were presented as follows: "Influenza," by Dr. F. H. Schneider, Ashland, Pa.; "Heaves," by Dr. I. C. Newhard, Ashland, Pa.; "Azoturia," by Dr. Otto G. Noack, Reading, Pa.; all being well discussed by those present.

The meeting adjourned to meet at Allentown on the third Wednesday in March, 1899.

E. D. Longacre, D.V.S.,
Secretary.

### VETERINARY MEDICAL ASSOCIATION OF NEW YORK COUNTY.

The regular monthly meeting was held at the Academy of Medicine, 17 W. Forty-third Street, January 4, 1899. President Dr. Robertson called the meeting to order at 8.30 P.M. The following members were present: Drs. Ryder, Hanson, MacKellar, Clayton, Bretherton, Bell, J. S. Cattanach, Ackerman, O'Shea, and Winslow; Dr. G. E. Griffen, of the Fifth United States Cavalry, being present as a visitor.

The Secretary being absent on account of sickness, Dr. C. E. Clayton was elected Secretary *pro tem.* Minutes of the previous meeting were read and approved.

The Board of Censors recommended that Dr. Lamkin's case be laid over until the next meeting; it was so ordered.

Dr. Hanson then read a valuable paper entitled "Digitalis and its Uses," [1] it being discussed by the members. The subject of "Azoturia" was then brought up for discussion.

Dr. G. E. Griffen reported a very interesting case of absence of rumination in a cow.

A vote of thanks was extended to Dr. Hanson for his paper.

By a vote of the members present the Constitution and By-laws were suspended and the Board of Censors was ordered to pass on the names of Dr. A. Liautard, for honorary membership, and Theodore Keller, graduate of A. V. C., 1892, for active membership. The Board reported favorably, and they were duly elected.

Dr. MacKellar, at the request of Dr. Gill, he being absent, read a letter from Dr. L. Pearson to Dr. Gill that, owing to a change in administration of the State of Pennsylvania, he would like a letter written to the Governor of said State recommending Dr. Pearson's reappointment. Moved by Dr.

[1] See page 67.

MacKellar that Dr. Gill be empowered to draft a letter and forward it to the Secretary, he to send it to the proper parties.

Dr. Hanson moved that a committee of three be appointed to draft resolutions urging the Governor to reappoint Dr. L. Pearson. The President appointed Drs. Hanson, MacKellar, and Ackerman. The bills of Clark and Zugalla and for rent ordered paid.

C. E. CLAYTON, D.V.S.,
Secretary pro tem.

### GENESEE VALLEY VETERINARY MEDICAL ASSOCIATION.

THE January meeting of this Association was held on Thursday, January 12th, at the Livingston Hotel, Rochester. The following members were present: A. Drinkwater, President; A. G. Tegg, Secretary; L. R. Webber, Treasurer; E. Knight and J. C. McKenzie, all of Rochester; W. G. Dodds, Vice-President, Canandaigua; T. S. Rich, Avon; O. B. French, Honoeye Falls; J. H. Taylor, Henrietta; A. Y. Earl, Palmyra; N. N. Lefler, Geneseo; J. Steiner, Bergen; L. J. Palmer, Sonyea; W. B. Switzer, Williamson; E. D. Burns, Fairport; P. J. Johnson, Sodus; G. C. Kesler, Holly; D. P. Webster, Hilton; E. H. Nodyne, Lyons; and T. Flood, Gorham.

The reports of the officers showed the Association to be in a prosperous condition.

Dr. A. L. Shaw, of Albion, was elected to membership.

After certain routine business had been transacted the following directors were elected for the ensuing year: Drs. Drinkwater, A. G. Tegg, W. G. Dodds, French, Knight, Webber, Steiner, Burns, Switzer, and Rich.

They elected from among their number the following officers: President, A. Drinkwater; Vice-President, W. G. Dodds; Secretary, Emil Knight; Treasurer, L. R. Webber, and the following censors: Drs. A. G. Tegg, O. B. French, J. Steiner, E. D. Burns, W. B. Switzer, and T. S. Rich.

The membership certificate was accepted as presented, and the committee, consisting of Drs. Knight, McKenzie, and Webber, was discharged.

A former action of the Association was reconsidered, resulting in the adoption of the red instead of the gold seal for the above certificates.

The Secretary was empowered to place the names of each member on the membership certificates, and to retain the same until ready for delivery.

The subject of illegal practicing was brought up, to which a number of the members made responses.

A motion was made and carried for members to send the names of all illegal practitioners in their district to the Secretary, and he to forward a copy of the letter, presented by one of the members, together with a printed transcript of the law, to such violators.

Papers were presented and read by the following:

Mr. J. B. Y. Warner, President of the Rochester Humane Society, concerning the work done and good accomplished by this society, concluding by asking our "friendship and co-operation in the work."

Dr. Nodyne: "Colic," confining himself most entirely to the spasmodic and flatulent varieties. He was more or less opposed to the use of opiates.

Dr. Kesler: "Deadly Nightshade, or Belladonna." He related his personal experience with cattle and sheep that had partaken of the same, with the symptoms as he found them.

Dr. McKenzie having a bad cold, his paper was read by the Secretary; "Influence of Mind on Diseases of the Body, or the Difference between Veterinary and Medical Science," among other things, dealing with the instinct of our patients against the brain faculties of the human patient, thereby accounting for imaginary diseases and ailments among the latter, and the failure of minute or homœopathic doses on the former.

All the above papers were listened to with great interest, and each brought forth considerable discussion.

A vote of thanks was tendered the writer in each instance.

The next regular meeting will be held some time during the second week in July, 1899.

EMIL KNIGHT, V.M.D.,
Secretary.

### VETERINARY MEDICAL ASSOCIATION OF NEW JERSEY.

THE fifteenth annual meeting of this Association was held at Trenton, January 12th. A large number of members were unable to be present on account of sickness, and after drafting resolutions on the death of Dr. James C. Dustan the meeting adjourned to reconvene at Newark on February 2d.

*Whereas*, It has pleased Almighty God to remove from our midst and from this Association our friend and brother, James C. Dustan; therefore, be it

*Resolved*, That while we bow in humble submission we recognize that we have sustained a great loss in that he was one of our most influential and highly respected members—one who had endeared himself to each and all by his gentlemanly deportment, kindly acts, and by his sense of honor and regularity in the practice of our honored profession.

*Resolved*, That we recognize our loss and the loss to the community in which he practised the profession of his choice, and we recommend to our fellow-members that they emulate the examples he set before us, and that they try to follow closely the precepts he always endeavored to teach and practise.

*Resolved*, That a copy of these resolutions be sent to the family of the deceased and be filed in full in the records of this Association.

W. B. E. MILLER,
L. P. HURLEY,
R. O. HASBROUCK,
J. W. HAWK,
S. LOCKWOOD,
Committee.

Meeting reconvened at the Arlington Hotel, Newark, on February 2d, at one o'clock, the President, Dr. W. H. Arrowsmith, in the chair. After a good dinner, proceeded with the regular business of the Association.

The election of officers resulted: President, Dr. L. P. Hurley; First Vice-President, R. O. Hasbrouck; Second Vice-President, H. W. Read; Secretary, S. Lockwood; Treasurer, B. F. King; Trustees, Drs. W. B. E. Miller, W. Runge, J. W. Hawk, J. M. Everitt, and M. M. Stage. All were elected for two years.

Dr. Arrowsmith made a lengthy address, and introduced Dr. Hurley, the

President-elect, who responded in a short and forcible address, and was followed by Drs. Miller and Hawk. It being late, Dr. Read, the essayist, asked to be excused from reading his paper, it being nearly time for his train. Excused.

Essayists for next meeting, Drs. Arrowsmith, Miller, and Hasbrouck.

Delegates to the New York Associations, Drs. Hawk and Lockwood; to the Pennsylvania Associations, Drs. Hurley, Hasbrouck, and Brown.

Adjourned to meet at same place in May.

S. Lockwood,
Secretary.

### NORTH CAROLINA VETERINARY MEDICAL ASSOCIATION.

The third annual meeting was held in the hall of the I. O. O. F., Pullen Building, Raleigh, December 23, 1898. The meeting was called to order at 12 m. by the Vice-President, Dr. T. B. Carroll, of Wilmington. The following members answered roll-call: Drs. H. G. Bessent, Durham; H. T. Baer, Greensboro; H. B. Murray, Hickory; Daniel Quinlivan, Wilmington; I. B. Ashecroft, Monroe; B. S. Griffin, Concord; I. M. Peden, Winston; W. H. Morris, Elizabeth City; L. H. Lambert, Asheville; J. W. Petty, Winston; W. C. McMackin, Raleigh.

Minutes of the last annual meeting were read and approved. Election of officers was called, resulting as follows: President, T. B. Carroll; First Vice-President, H. G. Bessent; Second Vice-President, G. H. Lambert; Secretary, J. W. Petty; Assistant Secretary and Treasurer, W. C. McMackin.

Drs. A. W. Goodwin, Raleigh; W. D. McMillian, Wilmington; and Mrs. J. W. Petty, Winston, were elected honorary members. The application for membership of Dr. Cooper Curtice, of New York, now a member of the faculty of the A. and M. College of Raleigh, was presented, and he was unanimously elected and a certificate of membership presented to him.

Dr. Curtice delivered an interesting address on "Texas Fever and Milk and Meat Inspection." He thought it was our duty as veterinarians to sound the alarm and press upon the health authorities the necessity of such legislation. By motion of Dr. McMackin, Dr. Curtice was appointed a committee of one, to be assisted by every member of the Association, to draft such a law on this subject as will be fitting to North Carolina.

Several communications were read and filed.

Report of the Secretary on finances was read and accepted.

A vote of thanks was unanimously voted to the Odd-Fellows for the use of their hall. Dr. Curtice was appointed on the Board of Examiners.

After a dinner, in which all participated, the meeting adjourned to meet during the summer at such time and place as may be named by the Secretary.

J. W. Petty,
Secretary.

W. C. McMackin,
Assistant Secretary and Treasurer.

### VETERINARY MEDICAL SOCIETY OF THE UNIVERSITY OF PENNSYLVANIA.

Meeting was called to order December 9, 1898, at 8 p.m. Mr. Gilbert was appointed critic. The programme of the evening consisted of an address by Prof. S. J. J. Harger, the subject being "Saline Transfusions." The subject was ably handled by Prof. Harger; he also demonstrated the method of injection. A vote of thanks was extended Prof. Harger.

The greater part of the meeting was consumed in transacting the business of the Association.

Meeting adjourned at 10.20 P.M.

Meeting was called to order January 15, 1899, at 8 P.M. This meeting was important from the fact that the officers for the second term were elected. The gentlemen honored on this occasion were Mr. Hoopes, '99, President; Mr. Miller, '99, Vice-President; Mr. Kerns, '99, Treasurer. Messrs. Jacobs, '99; Young, 1900; Stehle, 1900, and Walters, 1901, were elected to the Executive Committee.

Dr. Hoskins addressed the meeting, his subject being "Veterinary Legislation." His address was short, but was highly appreciated by all.

Meeting was called to order December 16, 1898, at 8.05 P.M. After transacting a few business matters the meeting adjourned to the University Restaurant, where the *Annual Banquet* was held. Among those present were Drs. J. W. Adams, S. J. J. Harger, W. Horace Hoskins, and Wm. G. Shaw. Owing to the absence of the Dean, Dr. Leonard Pearson, from town, he was unable to be present. We are happy to say that the banquet was a success and will go down in history as one of the enjoyable affairs of college life, as it carried out the old adage:

"A little nonsense now and then
Is relished by the wisest men."

L. A. NOLAN,
Secretary.

### MONTREAL VETERINARY MEDICAL ASSOCIATION.

A REGULAR meeting was held on Wednesday evening, January 18, 1899, in the library of the College, Dr. Baker presiding. There was a good attendance of members, supplemented by the presence of Drs. Sugden and Moore.

After roll-call and the reading of the minutes of the last meeting, the Chairman called upon Mr. McGregor for his case-report, which was one of "Perforation of the Abdominal Wall in a Three-year-old Colt." This animal was wounded in the abdominal parietes by some object (supposed to be a piece of wood or such like object), and, as the animal was a considerable distance from home, was not attended until the following day. Examination revealed a wound on the abdomen about two and a half by three inches in size, a few inches behind the last rib, somewhat inferolaterally, from which protruded peritoneum in a mass of about eight to ten inches in length. Having nothing better at hand, it was removed by a pair of scissors, and the hemorrhage arrested by cold water being freely applied. The wound was washed with a solution of corrosive sublimate, 1:1000, and the lower bowel relieved by enema.

Fever slight; gave febrifuge; ordered animal to be fed on soft diet. Dressed the wound twice a day with a carbolic solution. Next day abdomen began to swell, and continued doing so for four or five days, reaching as far as the sternum and extending well up the sides; swelling of an œdematous character; applied hot fomentation and poultice. About the fifth day pus began to flow from the wound, and continued to do so for two weeks. Dressed wound with carbolized oil. Swelling began to recede, and in four weeks the animal had made a complete recovery.

After some discussion on the case, the Chairman called upon Mr. Hammond for his essay on "Hog-cholera and Swine-plague." In this interesting essay Mr. Hammond clearly defined the difference existing between these two diseases, although to a great extent they resembled each other, as it required an examination of the internal organs after death to clearly distinguish between them, and, owing to the similarity of the two diseases, would not say what proportion of the loss entailed by these diseases could be attributed to the one and what to the other, as hog-cholera and swine-plague not only resembled each other in symptoms, but also in their effect on the bodies of the affected animals. Both were caused by germs, and both germs must be combated by such means to destroy them after they have been introduced upon the premises. Both diseases were infectious, and had to be treated along similar lines, and the agents used to destroy the germs of one would destroy the other. Mr. Hammond said that the other infectious diseases which sometimes attack hogs had never been introduced into this country, or have never approached in their destructive character the two diseases named. The erysipelas of the Continent of Europe appears to be the most fatal of the swine diseases in the countries where it is known, but has, however, never been recognized in America and probably never introduced into this country. Anthrax occasionally affected hogs, but was infrequent and confined to certain regions, and affected other animals besides. This and erysipelas were the only diseases liable to be mistaken for hog-cholera and swine-plague. The virus of hog-cholera was particularly more fatal to young pigs, was more tenacious and more resisting to the conditions which affect the vitality of bacteria than that of swine-plague, and was more easily spread and communicated to healthy animals. Period of incubation varied from four to twenty days, during which time the germs were multiplying slowly and overcoming the vital powers of the animal by their products. The bacilli of hog-cholera were slightly larger and more elongated than those of swine-plague, and were provided with flagellæ, which enabled them to move rapidly in liquids, while the germs of swine-plague had no such appendages, and were involuntarily moved about by the liquid in which they floated. The essayist went on at length to describe the differences between these two germs, their mode of invasion, and the different methods of producing them experimentally. After enumerating the symptoms common to the two diseases, Mr. Hammond said that although the symptoms were not noticeably different, yet frequently, however, the lungs were extremely inflamed in swine-plague, and in that condition the breathing is more oppressed and labored and the cough more frequent and prompt. The course of the disease varied from one to two days to two to three weeks. As to the diagnosis, he said that after enumerating the various symptoms presented we could readily come to a conclusion that either one or both diseases were present. The prognosis is unfavorable, especially if the animals were very susceptible and the contagion very virulent. The loss sometimes reached 95 to 100 per cent., but at other times if the animals were more capable of resisting the contagion, did not exceed 20 to 60 per cent., but what animals did recover from the disease often remained lean, stunted in growth, or never became really healthy hogs. As to the treatment of the disease, he said that the use of antitoxin serum appeared at present to be a much more promising method, combined with sanitary regulations, of diminishing the losses. The serum is prepared by inoculating horses and cattle with cultures of the diseased germs, and repeating those inoculations with gradually increasing doses until the animals have attained a high degree of immunity. The blood of such animals injected under the skin promises the power of curing such hogs and in preventing well ones from taking the disease. Unless the blood is to be used immediately after it is drawn, which is not often the case, it is allowed to coagulate, and the liquid portion or serum is separated and preserved for future use, so that we had every reason to believe that in the serum we had a practical method of preventing the greater part of the losses from these diseases. But it would have to be tried on a larger scale before absolute assurance could be given; he hoped that all doubts would be cleared up by the experiments for 1899. The essayist then enumerated the characteristic post-mortem lesions of the disease, concluding by drawing the attention to the fact that in hog-cholera the first effect of the disease was found to be on the intestines, with secondary invasion of the lungs, and in swine-plague the first effect was found to be on the lungs and the invasion of the intestines a subsequent process. A discussion ensued, with especial reference to the use of antitoxic serum as a prophylactic and curative remedy, it being finally conceded that the results so far obtained were of practical value.

The chairman then addressed the meeting with regard to the quarantine regulations and the methods of stamping out the diseases. He said that it was a well-established fact that ordinary treatment was useless, but hoped for good results from the use of antitoxin serum. In Canada the disease had never reached grave proportions, but it had been a scourge to some parts of the United States, and as they had the diseases before Canadians, we reaped some benefit in regard to the mode of combating them. He attributed our small loss to the rigorous methods the government had taken in regard to these diseases, and the owners of animals should co-operate with the government and aid in their suppression, concluding by thanking Mr. Hammond for his excellent paper.

Mr. Henderson was appointed essayist for the next meeting, and Mr. Gellalty to report a case, after which the meeting adjourned.

James McGregor,
Secretary and Treasurer.

### VETERINARY ASSOCIATION OF THE DISTRICT OF COLUMBIA.

The regular meeting was called to order January 29, 1899, by the Vice-President, Dr. D. E. Buckingham. Members present: Drs. Buckingham, Dawson, Gelston, Rome, Turner, and C. B. Robinson. Visitors: Dr. Nelson P. Hinkley, of Buffalo, N. Y., and several students of the United States College of Veterinary Surgeons. Minutes of previous meeting read and approved.

The unfinished business was the report of the Committee on Legislation. The chairman, Dr. Buckingham, reported that no action had yet been taken by Congress regarding the bill to regulate the practice of veterinary medicine in the District of Columbia. The Secretary reported that the bill was in the hands of the health officer for report, and the committee was instructed to call upon the health officer and make known the wants of this association regarding said bill.

Army veterinary legislation was brought up and discussed very fully by Drs. Turner and Robinson.

It seemed to be the consensus of opinion that while the provision in the Hull Army Reorganization bill giving the rank, pay, and allowances of second lieutenant of cavalry to the veterinarians, two of whom were allowed to each regiment of cavalry, was a great stride in the right direction, yet it would not meet the demands of the service, which required a corps of veterinarians headed by a competent man.

Dr. C. F. Dawson then read a very interesting and instructive paper on "Tuberculin and Mallein." The history of each of these animal toxins was given, together with a description of the characteristics of the two bacilli which produced them. The growths and their appearances on various media were described, and finally the technique of the production of each toxin were described and commented upon, and their value as diagnostic agents was discussed, together with the cause of this reaction in the live body. The members of the Society evinced a great interest in Dr. Dawson's paper, and upon its conclusion asked many questions regarding it.

Most of the members have used both of these toxins a great deal of late, and were greatly interested in the scientific presentation of the facts given in the paper. A vote of thanks was extended the doctor for his very able and interesting paper. Several members then took the floor, and told of their experience with both tuberculin and mallein.

Meeting adjourned.

J. P. Turner, V.M.D.,
Secretary.

### MINNESOTA STATE VETERINARY MEDICAL ASSOCIATION.

The fourth semi-annual meeting was held on January 12th and 13th at the State Experimental Farm, St. Anthony Park, St. Paul. A large number of the city and country members were present.

The reading of the Secretary's and Treasurer's reports were dispensed with for the time being, and the clinical programme proceeded with.

Dr. M. H. Reynolds presented a number of interesting cases of obscure lameness, among which was one of "Embolism of the Femoral Artery." A case of recovered osteoporosis was also presented, which had been cured by the daily administration of phosphate of lime. The same case was shown to the attending members at the meeting held a year ago, when the animal was suffering from a very pronounced attack of osteoporosis.

Dr. R. Price, of St. Paul, presented a case of a well-bred pointer dog which had been shot in the lumbar vertebræ, and as a result the nigh hind-leg remained in a condition of tonic spasm with the back badly arched. The dog was destroyed, and a minute dissection showed a blood-clot at the base of the brain.

Dr. C. C. Lyford, of Minneapolis, presented a case of deafness in a well-bred dog as a result of an injury. The doctor also made a very interesting dissection of a horse's head presenting extensive injuries to the roof of the mouth.

Dr. B. A. Pomeroy, of St. Paul, presented a chestnut gelding which had recovered from a very severe attack of purpura hemorrhagica, giving a full history and treatment of the case.

Dr. Reynolds gave a demonstration in caponizing.

The members then adjourned until after supper, when the meeting was called to order by Vice-President Reynolds. After the reading of the Secretary's and Treasurer's reports the Secretary read an address from President N. S. Erb, in which the latter expressed his sincere regrets at not being able to attend, and wishing the members a successful and prosperous meeting.

A lively discussion then followed on dealing with cases of illegal practice, and a number of important motions to that effect were made and carried.

The following members then reported some interesting cases: Dr. J. S. Butler, of Minneapolis, "Ossification of Thyroid Gland and Portion of Larynx Following the Operation for Roaring; "Retention of Pus in Sinuses Necessitating Trephining." Dr. C. C. Lyford, "Case of Tenotomy." Dr. W. Amos, of Owatonna, presented a fine specimen of a "Two-headed Calf," delivered by him a short time ago. Dr. J. N. Gould, of Worthington, reported an interesting operation for a "Tumor in a Cow." Dr. J. A. Hanisch, of Lake City, presented a "Tooth-deposit Found in an Abscess of the Thyroid Gland."

The nomination of officers then took place, and the following were elected for the ensuing year: President, Dr. M. H. Reynolds, of St. Anthony Park. First Vice-President, Dr. S. H. Ward, of St. Cloud. Second Vice-President, Dr. W. Amos, of Owatonna. Secretary and Treasurer, Dr. L. Hay, of Faribault. Board of Trustees, Drs. M. H. Reynolds, L. Hay, K. J. McKenzie, R. Price, and C. C. Lyford.

The meeting then adjourned until January 13th, when the following operations were demonstrated: "Cunean Tenotomy," Dr. K. J. McKenzie; "Anterior and Posterior Tibial Neurectomies for Relief of Spavin Lameness," Drs. S. D. Brimhall and K. J. McKenzie; "Arytenoideraphy," Dr. M. H. Reynolds; Dr. W. Amos acted as anæsthetist; "Ovaridectomy in a Bitch," Dr. J. J. Annand.

The meeting adjourned for dinner.

1.30 P.M. "Tenotomy," Dr. C. C. Lyford; "Cauterization of Tendons," Dr. B. A. Pomeroy; "Extracting the Third Lower Molar Tooth," Dr. J. P. Anderson; "Cauterizing Exostosis on Inside of Tibia," Dr. J. S. Butler; "A Dissection of Inguinal Region," Drs. Brimhall, McKenzie, and Annand; "High Plantar Neurectomy for Ringbone in Hind Leg," Dr. L. Hay; "Low and High Plantar Neurectomies for Navicularthritis," Dr. C. C. Lyford.

Meeting adjourned for supper.

At 7.30 a bay horse was cast, which was supposed to be a one-sided cryptorchid, and Dr. K. J. McKenzie proceeded to operate, when he found on each side of the scrotum cicatrices from former incisions. As the right testicle was down and the owner left word that the hidden testicle had not as yet been removed, the doctor naturally made his incision on the left side. However, after separating the fibrous thickening, he found what he pronounced to be the stump of the spermatic cord. The history of the case was then cleared up. Some time ago the horse had been cast for operation by an unskilful castrator, who removed the left testicle, which at that time was down, and made an incision on the right side in the attempt to get the right testicle, which, however, at that time was located high up in the inguinal canal, but had since descended. Such a case as this proved to be a very instructive one in cryptorchid castration, and confirmed the fact that the owner's word cannot be relied upon in these cases.

"Anterior and Posterior Tibial Neurectomies," Dr. M. H. Reynolds.

A vote of thanks was then tendered Drs. M. H. Reynolds and S. D. Brimhall for their courtesy and assistance in making this meeting such a successful one, after which the meeting adjourned.

L. HAY, V.S.,
Secretary and Treasurer.

### PENNSYLVANIA STATE VETERINARY MEDICAL ASSOCIATION.

THE annual meeting will be held at Veterinary Department, University of Pennsylvania, Thirty-sixth and Pine Streets, March 7 and 8, 1899.

On the evening of the first day Dr. Alfred Stengel will address those present on the "Importance of a Wholesome Meat Supply;" Dr. M. P. Ravenel, on "Some Bacterial Diseases of Animals Transmissible to Man;" Dr. Robert Formad will give some "Pathological Demonstrations;" Dr. John W. Adams will make some pertinent remarks in "Suggestions for the Improvement of the Meat-inspection Service of Philadelphia."

During the first day's session there will be demonstrations of methods of casting and securing animals for operations, operations for the relief of spavin, operative treatment for the prevention of cribbing, oöphorectomy of cow and bitch, operation for relief of spring-halt.

The election of officers will be held the first day, and the following programme will be carried out:

J. C. Michener, "Prognosis." A. N. Lushington, "Stock Farm Veterinary Practice as a Post-graduate Course." J. F. Butterfield, "Castration of Cryptorchids (ridglings)." W. S. Phillip, "Tracheotomy in Laryngitis and Choking." Otto G. Noack, "Azoturia." N. E. Reinhart, "Tuberculosis in Dairy Cattle, and How Shall We Get Rid of It." Leonard Pearson, "Dysentery in Calves." Robert G. Rice, "The Use of Chloral Hydrate in Indigestion and Colic." J. B. Irons, "Fracture of Tibia."

Luncheon will be spread each day at 1 P.M. in the assembly-room by courtesy of the Philadelphia veterinarians. A generous invitation is extended to all veterinarians to be present, and it is hoped that the Associations in adjoining States will send duly accredited delegates.

